# Density-dependent coral recruitment displays divergent responses during distinct early life-history stages

**DOI:** 10.1098/rsos.170082

**Published:** 2017-05-17

**Authors:** Christopher Doropoulos, Nicolas R. Evensen, Luis A. Gómez-Lemos, Russell C. Babcock

**Affiliations:** 1 CSIRO Oceans and Atmosphere, Dutton Park, Queensland 4102, Australia; 2 Marine Spatial Ecology Lab, Australia Research Council Centre of Excellence for Coral Reef Studies and School of Biological Sciences, The University of Queensland, St Lucia, Queensland 4072, Australia; 3 Griffith School of Environment, Australian Rivers Institute—Coast and Estuaries, Griffith University, Nathan, Queensland 4111, Australia

**Keywords:** Allee effect, density-dependence, recovery, recruitment, regulation, resilience

## Abstract

Population growth involves demographic bottlenecks that regulate recruitment success during various early life-history stages. The success of each early life-history stage can vary in response to population density, interacting with intrinsic (e.g. behavioural) and environmental (e.g. competition, predation) factors. Here, we used the common reef-building coral *Acropora millepora* to investigate how density-dependence influences larval survival and settlement in laboratory experiments that isolated intrinsic effects, and post-settlement survival in a field experiment that examined interactions with environmental factors. Larval survival was exceptionally high (greater than 80%) and density-independent from 2.5 to 12 days following spawning. By contrast, there was a weak positive effect of larval density on settlement, driven by gregarious behaviour at the highest density. When larval supply was saturated, settlement was three times higher in crevices compared with exposed microhabitats, but a negative relationship between settler density and post-settlement survival in crevices and density-independent survival on exposed surfaces resulted in similar recruit densities just one month following settlement. Moreover, a negative relationship was found between turf algae and settler survival in crevices, whereas gregarious settlement improved settler survival on exposed surfaces. Overall, our findings reveal divergent responses by coral larvae and newly settled recruits to density-dependent regulation, mediated by intrinsic and environmental interactions.

## 1. Introduction

The recruitment of new individuals is a key ecological process for the maintenance and recovery of natural populations. Following major disturbances, new individuals supplied from remnant populations recruit to newly available space, with their subsequent growth and survival driving population recovery [1–4]. But recruitment and population growth are not infinite, and there are density-dependent processes that regulate populations [5–7]. Population dynamics are (i) density-dependent when proportional growth is negative above a carrying capacity, (ii) inverse density-dependent when proportional growth is positive below a carrying capacity, or (iii) density-independent when proportional growth or loss do not respond to density. Inverse density-dependence can also lead to an Allee effect, where a population declines past a lower threshold and leads to extinction [5]. Hence, understanding how propagule density influences recruitment is necessary to quantify the thresholds needed for population recovery.

For many benthic marine invertebrates and fish, recruitment involves a bipartite life history with pelagic larvae and benthic recruits. Recruitment is thus a complex process, often divided into three major life-history phases involving (i) larval availability and transport, (ii) larval settlement ecology, and (iii) post-settlement ecology [8–10]. Each life-history phase can be considered a recruitment bottleneck, strongly influenced by bio-physical interactions [11–13]. In open, size-structured populations, early post-settlement survival is often considered the most severe bottleneck, given the minute size and high mortality of newly settled individuals [14–17]. Yet, the aggregative behaviour of marine fish and invertebrates as pelagic and settling larvae [10,18,19] means that density-dependence is a central contributor to demographic bottlenecks at both pre- and post-settlement life-history phases.

Interactions between intrinsic and environmental factors can change the strength and direction of density-dependent effects [20,21]. Inverse density-dependent settlement for benthic invertebrates and fish is often driven by high availability of settlement space and refugia, whereas competition and predation often cause direct density-dependent survival (e.g. [13,22]). This is particularly evident in coral reef fish, where density-dependence has been shown: (i) only in the presence of both resident-ambush and transient-pursuit predators, with survival becoming density-independent when only one type of predator is present [23]; or (ii) in the presence of only resident-ambush predators when the availability of refugia is limited and causes strong intraspecific competition [24]. For benthic marine invertebrates that attach themselves to the substratum, two-dimensional space is always ultimately limited. Settlement is often proportional to available space, with space limitation driving density-dependent and gregarious settlement [13,25,26]. Gregarious settlement increases post-settlement survival in some cases [27,28], mediated by size-escape mechanisms, but not others [29]. At low settlement densities, survival is often density-independent [25,26,30], but at high densities, gregarious settlement can also attract predators and increase post-settlement mortality [25].

Coral populations are often in a state of recovery, making degraded coral reef ecosystems particularly vulnerable to Allee effects due to habitat and population fragmentation that act on coral recruitment [31]. Bottlenecks to coral recovery can be driven by limited larval supply [32,33], environmental factors such as refugia, predation (direct or indirect), grazing and competition that interact with settlement and post-settlement stages [12,34,35], or a combination of both. Surprisingly, only a limited number of studies have investigated the role of density-dependent effects on coral recruitment processes. Patterns reveal that: (i) larval survival is negatively related to density in *Montipora capitata* [36]; (ii) larval settlement increases with larval density using mass *in situ* culturing with gametes collected from spawning slicks [37], *Acropora digitifera* [38], and *Acropora muricata* and *Acropora tenuis* [39]; (iii) larval settlement is positively related to crustose coralline algae (CCA) but negatively related to turf algae cover with *Siderastrea radians* [13]; and (iv) the relationship between coral settlers and recruits is density-dependent so that increasing settlement density does not improve recruit densities in *Acropora* spp. [38,39]. Yet, no study has conducted a systematic evaluation of density-dependent effects at each major early life-history stage for a single species, and mechanistic understandings are vague. Hence, we used three complementary experiments aimed to examine how intrinsic and environmental factors can drive density-dependent responses during coral larval survival, larval settlement and post-settlement survival in *Acropora millepora*. Experiments examining density-dependent regulation of larval survival and settlement were conducted in a laboratory and excluded any environmental interactions, while the experiment examining post-settlement survival was conducted in a field setting and included interactions with environmental factors.

## 2. Material and methods

### 2.1. Study location and coral larvae culturing

This study was conducted at Coral Bay Research Station and nearby reefs, Ningaloo Reef, Western Australia, from March until May 2016. Eight gravid colonies of the common branching coral *A. millepora* were collected from shallow reefs (approx. 3 m depth) in Coral Bay (23.173984° S, 113.760459° E) and transported to the local jetty for access during spawning. Colonies were isolated in 60 l tubs at sunset and all spawned on the 2nd of April from 21.00 to 22.00, 10 days after the full moon. The egg–sperm bundles were collected and transported to the research station where they were transferred into two 20 l tubs of filtered seawater. Water agitation broke apart the bundles to promote cross-fertilization. Fertilization success was periodically monitored using a dissecting microscope, and after 3 h more than 50% of subsampled eggs were fertilized and had undergone division. Eggs were removed from the top of the water column and placed into large outdoor 200 l sumps for rearing with filtered seawater and aeration. Half water changes took place every 6 h for the first 48 h, and every 24 h thereafter. Similarly, aeration was low for the first 48 h and was increased thereafter. All seawater used for fertilization and rearing was filtered using a four-stage canister stack and UV sterilization (Odyssea CFS-1000). The mean temperature of the seawater during the larval culturing period was 24.9°C (±0.9 s.d., min = 22.5°C, max = 26.8°C).

### 2.2. Experimental overview

Three experiments were conducted to test density-dependent responses of larval survival, larval settlement and post-settlement survival. The two experiments investigating larval survival and larval settlement were conducted in laboratory conditions that excluded variations in environmental factors, isolating the effects of larval density only. By contrast, the experiment investigating post-settlement survival was conducted using tiles that incorporate microhabitat rugosity and were transplanted onto reefs, incorporating the effects of density interacting with environmental factors (i.e. refugia, competition and predation).

### 2.3. Experiment 1: density-dependent larval survival

We began an experiment to examine the effect of larval density on larval survival once the coral larvae had developed into swimming planulae, 60 h following spawning. *Acropora millepora* planulae are lecithotrophic (i.e. non-feeding), and non-feeding *Acropora* planulae have been shown to remain competent in laboratory conditions for at least six weeks following spawning [40]. Swimming and healthy coral larvae were sampled and placed into sterile 20 ml glass containers (scintillation vials) with filtered seawater (0.2 µm and UV). Previous work has shown that coral larvae are found at maximum densities of approximately five individuals per 20 ml in multi-species spawning slicks [41], so we chose densities that ranged below and above this maximum. Larvae were stocked at 1, 3, 6, 10 and 20 individuals per 20 ml, with five replicate containers per density. At 60, 85, 107, 130, 154, 210, 263 and 288 h following spawning, the number of swimming and dead larvae were individually counted and surviving larvae transferred into new filtered seawater. Larvae were recorded as alive if they were observed (by eye) swimming, but if not moving they were assessed under a dissecting microscope to verify whether they were dead or alive. The experiment took place in a laboratory at 26°C.

Larval survival data were analysed using two approaches. First, trends in larval survival at the different densities were investigated using a random-effects Cox proportional hazard model (Coxme) with the ‘coxme’ package [42] in R, with containers treated as a random effect. The model is a time-to-event analysis that allows for the stochastic rate at which an event occurs to vary, but makes a proportional hazards assumption that the relative effect among treatments is consistent over time. Second, because time had no obvious effect on larval survival rates (Coxme, *p* = 0.567), a subsequent analysis investigated the effect of larval density on proportional larval survival 12 days after spawning using a generalized linear mixed effects model (GLMM) with binomial error structure. Larval survival was the binomial response variable, initial larval density the continuous predictor and replicate containers incorporated as a random effect.

### 2.4. Experiment 2: density-dependent larval settlement

A complementary experiment was then conducted to examine the effect of larval density on larval settlement once the coral larvae were competent to settle, 7 days following spawning. Larvae were stocked at 1, 3, 6, 10, 20 and 50 individuals per 20 ml, with five replicate containers per density. Experimental settlement assays generally followed the procedures of Heyward & Negri [43], explained below. Swimming and healthy planulae that were searching and testing the substrata for settlement were sampled and placed into sterile 20 ml polystyrene cell culture wells with filtered seawater (0.2 µm and UV). The CCA *Porolithon onkodes* was used as a settlement inducer. *Porolithon onkodes* was collected using chisel and hammer from nearby reefs at a depth of approximately 1 m, fragmented into equally sized 5 × 5 mm chips, the thalli were cleaned with a toothbrush and tweezers under a dissecting microscope and the dead carbonate underside fraction of the chip was scraped off so that only the very thin surface layer of the CCA remained. Upmost care was taken to standardize the CCA chip size to 5 × 5 mm to remove any potential confounding interaction between larval density and CCA chip size. A single chip was placed in each well with the larvae.

The experiment took place in a laboratory at 26°C for 24 h from the time the larvae and CCA were introduced into the wells. Settlement was then scored by directly counting all larvae that had attached and metamorphosed in each well on the CCA chip and container surfaces under a dissecting microscope. The effect of larval density on proportional larval settlement was analysed using a GLMM with binomial error structure, with larval settlement as the response variable, initial larval density the continuous predictor, and replicate containers incorporated as a random effect.

### 2.5. Experiment 3: density-dependent post-settlement survival

A final experiment then took place to understand effects of gregariousness, initial settler density and competition on post-settlement survival. The experiment used a field-based approach, and generally followed procedures described in Doropoulos *et al*. [12]. Larvae were settled onto 10 × 10 cm settlement tiles made from a mix of calcium carbonate sand and cement at a ratio of 4 : 1. The tiles are a custom design that incorporate microhabitat complexity, with 24 crevices (total area = 54.8 cm^−2^ per tile) and 24 exposed surfaces (total area = 34.6 cm^−2^ per tile), previously shown to characterize coral recruitment on the reef benthos [12]. Tiles were preconditioned for a month at a shallow reef (approx. 3 m depth) in Coral Bay to develop a microbial and encrusting community important to coral settlement [43]. Upon retrieval, tiles were gently cleaned using toothbrushes to remove any turf algae, encrusting fleshy algae (EFA), foliose macroalgae and heterotrophic invertebrates, and were placed with the competent coral larvae in the rearing tub at 6 days after spawning. Corals that settled onto the tiles were scored after 2–3 days using a dissecting microscope. Only individuals that settled on the upward facing exposed and crevice microhabitats were included, with all other individuals scraped off including those on tile undersides and vertical edges (electronic supplementary material, figure S1).

Each settler was mapped according to its location on the tile and gregariousness (i.e. whether an individual was settled touching another individual or not). Tiles were then out-planted to five replicate reef-flat sites at 2–3 m depth, each separated by more than 8 km, with five tile replicates per site. Tiles were attached 2 cm above the reef substrate using base plates following the technique of Mundy [44]. Three extra tiles were mapped and used as a handling control by transporting them to the field sites during deployment, returning them to the laboratory the same day after 14 h and rescoring them. Controls showed that transportation of the experimental tiles to the field did not affect settler survival with less than 3% change in abundances. For the experimental tiles, all settlers were rescored *in situ* by a single observer (C.D.) following a 30-day period. The location and gregariousness were recorded over the previously drawn maps, indicating the outcome (mortality/survival) for every coral settler.

To assess the influence of competition on post-settlement survival, photographs of the tiles were taken *in situ* to quantify the community covering the settlement tiles. A grid of cells that corresponded to every exposed and crevice microhabitat was then overlaid onto the image of each tile and proportional cover of the different substrata within a cell estimated. Thus, the substrata in the immediate area surrounding any coral settler were known. Substrata were categorized as biofilmed tile, CCA, turf algae (Turf), sediment, EFA, foliose macroalgae (MA) and heterotrophic invertebrates (ascidians, bryozoans, sponges).

Analysis of the field experiment was conducted in multiple stages to understand (i) the distribution of corals between microhabitats (exposed, crevice) upon settlement and 30 days post-settlement; (ii) the community composition between microhabitats at 30 days post-settlement; and (iii) the effect of microhabitat, settler density, gregariousness (single, aggregated) and competition on the post-settlement survival of newly settled corals at 30 days post-settlement. First, to investigate the distribution of coral settlers between microhabitats, settler density was standardized to the number of individuals per square centimetre to normalize for the greater space availability in crevices compared with exposed surfaces. The same approach was applied to coral recruits at 30 days post-settlement. Paired *t*-tests were conducted to statistically test for any differences in the density of settlers or recruits between microhabitats, incorporating the correlation structure between crevice and exposed microhabitats within a tile. Second, multivariate community cover of the dominant groups found on the settlement tiles at 30 days post-settlement was then tested between microhabitats using a mixed effects model, with sites incorporated as a random effect. The multivariate analysis was conducted on a Bray–Curtis similarity matrix, using 999 permutations to generate *p-*values, on the raw data that were homogeneous without transformation (tested using permDISP).

The final analysis on post-settlement survival then used a step-wise modelling approach. Initial analyses showed significant interactions between microhabitats × gregariousness and microhabitats × density on coral post-settlement survival. Considering that benthic communities were also distinct between the two microhabitats (see Results), two separate analyses were conducted that investigated the effects of gregariousness, settler density and competition on post-settlement survival within each microhabitat. For each microhabitat, the original GLMM included the survival of every settler as the binomial response variable, gregariousness (single, aggregated) as a categorical predictor, initial settler abundances as a continuous predictor, cover of the seven substrata in a cell surrounding the settlers as continuous predictors, and site and tile replicates as random effects with tile replicate nested in site. All continuous variables were centred by their mean to make their effects comparable. Model simplification was then conducted using backwards elimination of predictors, comparing full and reduced models using likelihood ratio test (LRT, *χ*^2^) *p*-values. For exposed microhabitats, the best fit model incorporated settler survival as a function of gregariousness, and the cover of CCA, turf, EFA and sediment. For crevice microhabitats, the best fit model included settler survival as a function of settler density, and the cover of CCA, turf, EFA, sediment and MA. Both GLMMs had site and tile replicates as random effects with tile replicates nested in site.

All GLMMs were conducted using ‘lme4’ [45], *post hoc* tests of categorical interactions used ‘multcomp’ [46], and paired *t*-tests used the ‘stats’ package in R [47]. Fits of binomial GLMMs were estimated using ‘blemco’ [48], and dispersion was not found to be problematic for analyses of larval settlement and post-settlement survival. However, for larval survival, data were overdispersed, so an observation level random factor was added to the model. Multivariate analysis of the tile community was conducted in PRIMER with the perMANOVA extension [49].

## 3. Results

### 3.1. Density-dependent larval survival

**Figure 1.**
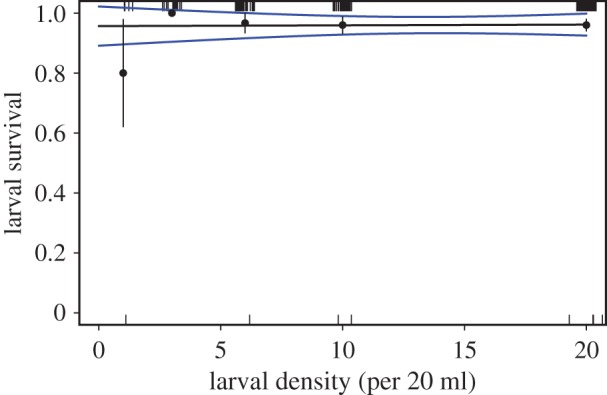
Relationship between larval density and proportional survival of *A. millepora* coral larvae 226 h following spawning. Experiment began 60 h following spawning. The black solid line represents the mean model fit, blue lines represent the s.e.m. of the model fit, black circles and vertical bars represent the mean and s.e.m. among tanks, and the small notches (top and bottom *x*-axes) represent individual data points. There were five replicate tanks per larval density.

No relationship was observed between initial larval density and larval survival from 2.5 to 12 days following the spawning of the *A. millepora* (figure 1 and table 1*a*; electronic supplementary material, figure S2). Thus, coral larval survival was density-independent and averaged greater than 80% for densities ranging from 3 to 20 larvae per 20 ml.

**Table 1.** Summary of generalized (binomial) linear mixed effects models testing density-dependent effects on coral larvae (a) survival and (b) settlement, and (c) post-settlement survival. The directions of effects are indicated, as well as statistical model details and outcomes. +, positive relationship; –, negative relationship; ∼, no relationship. LRT, likelihood ratio test. Italic text indicates effects with *p* < 0.1.

recruitment stage	response	predictor	direction	LRT	*p*-value
(a) larvae^a^	survival	initial density	∼	0.016	0.899
(b) settlement^a^	settlement	*initial density*	*+*	*3.367*	*0.066*
(c) post-settlement^b^	survival	*micro.*^c^* × greg.*^d^		*7.561*	*0.006*
		*micro.*^c^* × density*		*3.260*	*0.071*
exposed microhabitat		*gregariousness*	*+*	*10.013*	*0.002*
		CCA^e^	+	2.025	0.155
		turf	–	1.631	0.201
		EFA^f^	–	1.044	0.307
		sediment	–	2.240	0.134
crevice microhabitat		*initial density*	*−*	*7.238*	*0.007*
		CCA^e^	+	2.316	0.128
		*turf*	*−*	*7.728*	*0.005*
		EFA^f^	∼	0.031	0.860
		sediment	**–**	1.017	0.313
		macroalgae	∼	0.076	0.782

^a^Five tank replicates per density.

^b^Five site replicates; five tile replicates per site.

^c^Microhabitat = categorical with two levels (exposed, crevice).

^d^Gregariousness = categorical with two levels (single, aggregated).

^e^CCA, crustose coralline algae.

^f^EFA, encrusting fleshy algae.

### 3.2. Density-dependent larval settlement

**Figure 2.**
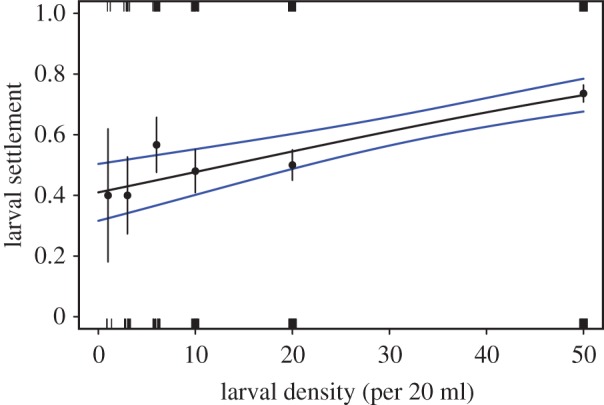
Relationship between larval density and proportional settlement of *A. millepora* after 24 h in settlement experiments conducted 7 days following spawning. See figure 1 legend for description of the symbols in the plots. There were five replicate tanks per larval density.

There was a positive relationship between the initial density of *A. millepora* and the probability of settlement (figure 2). Settlement ranged from 40 to 50% at densities of 1–20 larvae per 20 ml, increasing to greater than 70% at the highest density of 50 larvae per 20 ml. Variability among replicate containers within larval density was high, especially at lower densities (figure 2), resulting in a weak overall effect between settlement and density (LRT, *p* = 0.066; table 1*b*).

### 3.3. Density-dependent post-settlement survival

**Figure 3.**
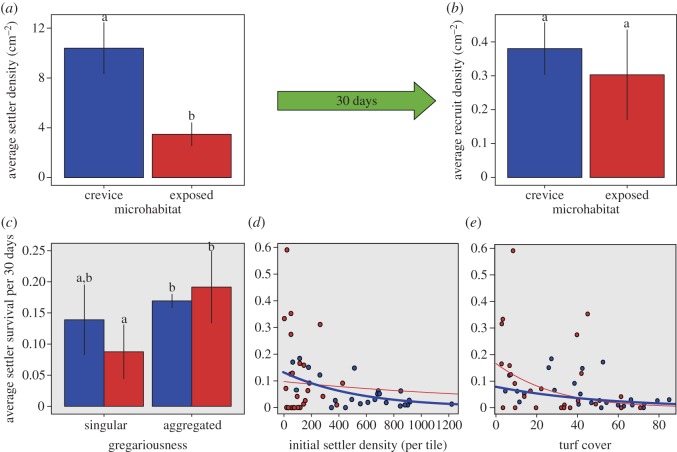
Average *A. millepora* densities (cm^−2^) among crevice (blue) and exposed (red) microhabitats at (*a*) settlement and (*b*) following 30 days in field experiments. Explanatory variables of post-settlement survival are displayed in grey panels and include the effects of (*c*) gregariousness, (*d*) initial settler density and (*e*) turf cover. Bars with different letters above them in *a*, *b* and *c* are significantly different, and solid lines in *d* and *e* represent the mean prediction of significant (thick and blue) and non-significant (thin and red) model fits. There were five replicate sites with five replicate tiles per microhabitat. Note the different scales for *y*-axes.

When competent larvae were provided with complex substrata, a total of 17 241 *A. millepora* settlers were mapped from 25 tiles, with a strong preference to settle in crevices compared with exposed microhabitats. There were 3.0 times more settlers in crevices compared with exposed surfaces (paired *t*-test, *p* < 0.001), with a mean ± s.e.m. of 10.39 ± 1.13 settlers cm^−2^ versus 3.48 ± 0.64 settlers cm^−2^, respectively (figure 3*a*). Subsequently, the proportion of gregarious settlement was higher in crevices than exposed microhabitats (59.7 ± 3.0% versus 48.1 ± 4.7%). However, just 30 days following settlement, average recruit density was the same in both microhabitats (paired *t*-test, *p* = 0.47), averaging 0.38 ± 0.06 recruits cm^−2^ in crevices and 0.30 ± 0.10 recruits cm^−2^ on exposed surfaces (figure 3*b*).

**Figure 4.**
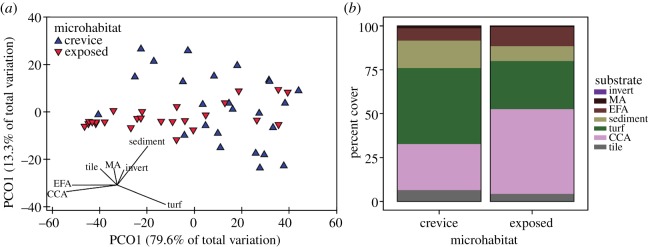
(*a*) Principal coordinate analysis and Pearson correlation vector of the substrata found in crevices (upward facing blue triangles) and exposed (downward facing red triangles) microhabitats on the settlement tiles 30 days following deployment. (*b*) Relative percent cover of each substrate found in crevice and exposed microhabitats on the settlement tiles 30 days.

The community found on the settlement tiles 30 days following deployment also differed between exposed and crevice microhabitats (PERMANOVA, *p* = 0.024; figure 4*a*). On exposed surfaces, CCA was dominant (48% cover) over turf algae (27%) followed by EFA cover (11%; figure 4*b*). A contrasting pattern was found in crevices, with turf algae dominating (43% cover) over CCA cover (26%), followed by sediment (16%) and EFA cover (7%; figure 4*b*).

Contrasting responses to intrinsic and environmental factors drove differential coral settler mortality between the microhabitats. First, gregarious settlement behaviour increased survival by greater than two times on exposed surfaces but did not improve post-settlement survival in crevices (figure 3*c* and table 1*c*). On exposed surfaces, survival of singular settlers averaged only 9% after 30 days compared with 19% for aggregated settlers. Second, a nonlinear negative relationship was observed between settler density and proportional survival of settlers found in crevices (figure 3*d* and table 1*c*). Survival averaged 13–10% for the lowest densities less than 200 settlers per tile, decreased to 6% and 4% for densities of 400 and 600 settlers and was lowest at 3 to 1% for densities ranging from 800 to 1200 settlers per tile. By contrast, there was no relationship between settler density and survival on exposed microhabitats, with highly variable survival occurring at densities less than 200 settlers per tile (figure 3*d*). Finally, of all the potential competitors, only increasing turf algae had a significant negative effect on post-settlement coral survival and this only occurred in crevice microhabitats (figure 3*e* and table 1*c*).

## 4. Discussion

While it has previously been shown that density-dependence has varying effects during coral recruitment that depends on the life-history phase and taxonomic identity [13,36–39], no previous work had conducted a systematic study to mechanistically understand how density-dependence influences recruitment success during each major early life-history stage in a single species. Our series of experiments demonstrates the complexities of density-dependent effects on coral recruitment, from the survival and behaviour of swimming and settling larvae isolated in laboratory settings, to ecological interactions that drive early post-settlement survival up to one month following settlement. Larval survival in the experimental arena free of competitors, predators or microbes was extremely high and density-independent from 2.5 days until 12 days following spawning. Using the same experimental arena, larval settlement displayed a weak positive response to increasing larval density due to the gregarious settlement behaviour of marine invertebrates. When offered settlement substrata with microhabitat rugosity, larval settlement was three times higher in crevice microhabitats that offer refugia from predators compared with exposed surfaces. However, settler survival was density-dependent in those crevice microhabitats but density-independent on exposed surfaces. Ultimately, the contrasting density-dependent and -independent survival of settlers led to similar densities of coral recruits in both microhabitats just one month following settlement, supporting the findings of Suzuki *et al*. [39] and Edwards *et al*. [38] that very high densities of settled larvae do not improve coral recruitment.

Propagule supply is the key first step to population recovery, and for coral reefs the supply of coral larvae is critical for habitat recovery following disturbances. Owing to their minute size, direct estimates of the actual supply of coral larvae and larval survival remain a mystery on reefs [50]. Hence, density-dependent thresholds for ecologically relevant abundances of coral larvae that can contribute to recruitment on reefs are unknown, but laboratory experiments can partly provide the information necessary for application to predictive modelling (e.g. [51,52]). Apart from this study where we found extremely high and density-independent survival of *Acropora* larvae (greater than 80%) from 2.5 days until 12 days following spawning, only one previous study has addressed the relationship between coral larval density and survival and found strong density-dependent survival of *Montipora* larvae from 2 to 7.5 days following spawning [36]. However, those experiments by Vermeij *et al*. [36] had densities of around 250 to 11 250 larvae l^−1^, well above the maximum of 230 larvae l^−1^ found in spawning slicks [41] and the maximum of 1000 larvae l^−1^ used in our study. Notably, similarly high rates of survival to our study were found with *Montipora* larvae at densities less than 1250 larvae l^−1^ [36]. Other studies using *Acropora* spp. at densities of 1000–1500 larvae l^−1^ have found survival ranges from less than 50% [51] and 15–90% [53] in the first 12 days following spawning, showing that larval survival is highly variable even in controlled laboratory conditions. Future experiments that incorporate refugia and predators with varying densities of coral larvae in laboratory settings are needed to more thoroughly examine whether the density-independent response observed in this study remains when environmental interactors are present. In addition, density-dependent responses may occur in the earliest stages following spawning when positively buoyant developing embryos are distributed predominantly on the water surface in the highest densities in spawning slicks.

Upon arrival of larvae to a patch of reef, the transition from the plankton to the benthos is the next key step in recruitment. Experiments that supersaturate larval densities *in situ* have found that settlement is enhanced compared with controls [37–39]. Our laboratory study supports these previous field experiments, showing that increasing larval density increases settlement rate, but here we also show that the relationship is not proportional and there appears a minimum threshold beyond which settlement is maximized. To illustrate, settlement rates were 50% at 20 larvae per 20 ml (and no different to the lower larval densities), but 75% at 50 larvae per 20 ml, resulting in a nonlinear increase in the abundance of settlers at the highest density. Suzuki *et al*. [39] also found no difference in settlement between low (25 larvae l^−1^) and medium (120 larvae l^−1^) larval densities, but significantly higher settlement at the highest larval density (600 larvae l^−1^), although the difference between the number of settlers was relatively proportional [39]. The positive relationship between larval density and settlement probability was statistically weak in our settlement assays (table 1*b*), due to the high among tank variability associated with settlement at the lower larval densities (especially 1 and 3 larvae per 20 ml). High settlement variability at lower larval densities is expected considering that invertebrate settlement is facilitated by positive chemical cues derived from conspecifics [19], and aggregative settlement of *A. millepora* has previously been shown in experimental studies [29]. It is also interesting that the variability of settlement rate declined at higher densities because more consistent or less variable recruitment is an important attribute when considering the resilience of a community or population following disturbance [50]. At the settlement stage of recruitment, nonlinear increases in settler abundances resulting from increasing larval densities support the notion that larval densities need to surpass minimal thresholds to achieve settlement success and avoid potential Allee effects on population recovery [5,31].

Microhabitat suitability for coral settlement is driven by known physical and chemical cues in marine invertebrates, and, similar to previous work with coral larvae [12,54,55], *A. millepora* larvae preferentially settled within crevices rather than exposed surfaces when provided with complex substrata in this study. However, in contrast with previous work [12,55–58], the refugia provided by crevices did not enhance post-settlement survival compared with exposed surfaces, averaging only 3.7% survival in crevices compared with 8.6% on exposed surfaces for the 30-day post-settlement period. In crevices, there was an overwhelming nonlinear negative relationship with increasing settler density, which was not apparent on exposed surfaces where post-settlement survival was density-independent. Moreover, nonlinear negative competitive interactions were also found with turf algae in crevices, exemplifying the influence of negative competitive effects found in cryptic microhabitats between turf algae and coral recruits [34,55]. No competitive effects were found to influence settler survival on exposed surfaces, but gregarious settlement doubled post-settlement survival compared with individual settlement. While we did not directly quantify predation in our field experiment, we infer the relationship between size-escape mechanisms and indirect predation by herbivorous fish drove the positive response. On exposed surfaces, turf abundance was reduced and CCA abundance was increased, most likely a result of herbivorous fish grazing [12,34,55,59]. Subsequently, the action of herbivorous fish grazing may have indirectly targeted individual coral settlers because indirect predation by herbivorous fish occurs on the smallest coral recruits, which are quickly avoided as their size increases [12,35,60,61]. The alternative hypothesis that aggregative settlement increases post-settlement mortality by attracting predators, as seen at high-densities of barnacle [25] and coral [62–64] recruits, is not supported by the results found in this study.

Collectively, our study shows that both excessively high and low densities of larvae and settlers are likely to contribute little to population recovery. Within the context of coral reef degradation (e.g. [31]), low larval supply causing recruitment limitation from bottlenecks at settlement appear the first risk to coral recovery. On the other hand, excessively high larval supply that results in supersaturated settlement densities will not contribute to the recruitment of individuals that survive early post-settlement bottlenecks, and thus promote population recovery, because of density-dependent regulation [38,39,65]. Hence, while successful coral recruitment and population recovery are likely to be optimal with consistent supplies of larvae to disturbed patches of reef without space limitation, the density threshold that enhances recruitment success remains elusive and requires further investigation. Importantly, regulators of early recruitment success can act at very local (e.g. microhabitat) to regional (e.g. meta-population larval connectivity) scales, interacting with multiple stressors, so the use of generic rules to model recovery dynamics of coral populations needs to be applied conservatively within a context-specific approach.

## Supplementary Material

Figure S1

## Supplementary Material

Figure S2

## Supplementary Material

Table S1

## Data accessibility

The datasets supporting this article have been uploaded as part of the electronic supplementary material, table S1.

## References

[1] Connell JH, Keough MJ 1985 Disturbance and patch dynamics of subtidal marine animals on hard substrata. In The ecology of natural disturbance and patch dynamics (eds Pickett STA, White PS), pp. 125–152. Orlando, FL: Academic Press.

[2] Young TP, Petersen DA, Clary JJ 2005 The ecology of restoration: historical links, emerging issues and unexplored realms. Ecol. Lett. 8, 662–673. (doi:10.1111/j.1461-0248.2005.00764.x)

[3] Nathan R, Muller-Landau HC 2000 Spatial patterns of seed dispersal, their determinants and consequences for recruitment. Trends Ecol. Evol. 15, 278–285. (doi:10.1016/S0169-5347(00)01874-7) 10856948 10.1016/s0169-5347(00)01874-7

[4] Doropoulos C, Ward S, Roff G, González-Rivero M, Mumby PJ 2015 Linking demographic processes of juvenile corals to benthic recovery trajectories in two common reef habitats. PLoS ONE 10, e0128535 (doi:10.1371/journal.pone.0128535) 26009892 10.1371/journal.pone.0128535 PMC4444195

[5] Courchamp F, Clutton-Brock T, Grenfell B 1999 Inverse density dependence and the Allee effect. Trends Ecol. Evol. 14, 405–410. (doi:10.1016/s0169-5347(99)01683-3) 10481205 10.1016/s0169-5347(99)01683-3

[6] Caley MJ, Carr MH, Hixon MA, Hughes TP, Jones GP, Menge BA 1996 Recruitment and the local dynamics of open marine populations. Annu. Rev. Ecol. Syst. 27, 477–500. (doi:10.1146/annurev.ecolsys.27.1.477)

[7] Hixon MA, Johnson DW 2009 Density dependence and independence In *Encyclopedia of Life Sciences (eLS)*. Chichester, UK: John Wiley & Sons, Ltd (doi:10.1002/9780470015902.a0021219)

[8] Ritson-Williams R, Arnold SN, Fogarty ND, Steneck RS, Vermeij MJ, Paul VJ 2009 New perspectives on ecological mechanisms affecting coral recruitment on reefs. Smithson. Contrib. Mar. Sci. 38, 437–457. (doi:10.5479/si.01960768.38.437)

[9] Pineda J, Reyns NB, Starczak VR 2009 Complexity and simplification in understanding recruitment in benthic populations. Popul. Ecol. 51, 17–32. (doi:10.1007/s10144-008-0118-0)

[10] Pawlik JR 1992 Chemical ecology of the settlement of benthic marine-invertebrates. Oceanogr. Mar. Biol. 30, 273–335.

[11] Wahle R, Steneck R 1991 Recruitment habitats and nursery grounds of the American lobster *Homarus americanus*: a demographic bottleneck? Mar. Ecol. Prog. Ser. 69, 231 (doi:10.3354/meps069231)

[12] Doropoulos C, Roff G, Bozec Y-M, Zupan M, Werminghausen J, Mumby PJ 2016 Characterizing the ecological trade-offs throughout the early ontogeny of coral recruitment. Ecol. Monogr. 86, 20–44. (doi:10.1890/15-0668.1).

[13] Vermeij MJA, Sandin SA 2008 Density-dependent settlement and mortality structure the earliest life phases of a coral population. Ecology 89, 1994–2004. (doi:10.1890/07-1296.1) 18705385 10.1890/07-1296.1

[14] Werner EE, Gilliam JF 1984 The ontogenetic niche and species interactions in size-structured populations. Annu. Rev. Ecol. Syst. 15, 393–425. (doi:10.1146/annurev.es.15.110184.002141)

[15] Gosselin LA, Qian PY 1997 Juvenile mortality in benthic marine invertebrates. Mar. Ecol. Prog. Ser. 146, 265–282. (doi:10.3354/meps146265)

[16] Hunt HL, Scheibling RE 1997 Role of early post-settlement mortality in recruitment of benthic marine invertebrates. Mar. Ecol. Prog. Ser. 155, 269–301. (doi:10.3354/meps155269)

[17] Rice JA, Miller TJ, Rose KA, Crowder LB, Marschall EA, Trebitz AS, DeAngelis DL 1993 Growth rate variation and larval survival: inferences from an individual-based size-dependent predation model. Can. J. Fish. Aquat. Sci. 50, 133–142. (doi:10.1139/f93-015)

[18] Selkoe KA, Gaines SD, Caselle JE, Warner RR 2006 Current shifts and kin aggregation explain genetic patchiness in fish recruits. Ecology 87, 3082–3094. (doi:10.1890/0012-9658(2006)87[3082:CSAKAE]2.0.CO;2) 17249233 10.1890/0012-9658(2006)87[3082:csakae]2.0.co;2

[19] Burke RD 1986 Pheromones and the gregarious settlement of marine invertebrate larvae. Bull. Mar. Sci. 39, 323–331.

[20] Hixon MA, Pacala SW, Sandin SA 2002 Population regulation: historical context and contemporary challenges of open vs. closed systems. Ecology 83, 1490–1508. (doi:10.2307/3071969)

[21] White JW 2007 Spatially correlated recruitment of a marine predator and its prey shapes the large-scale pattern of density-dependent prey mortality. Ecol. Lett. 10, 1054–1065. (doi:10.1111/j.1461-0248.2007.01098.x) 17692098 10.1111/j.1461-0248.2007.01098.x

[22] White JW, Samhouri JF, Stier AC, Wormald CL, Hamilton SL, Sandin SA 2010 Synthesizing mechanisms of density dependence in reef fishes: behavior, habitat configuration, and observational scale. Ecology 91, 1949–1961. (doi:10.1890/09-0298.1) 20715614 10.1890/09-0298.1

[23] Hixon MA, Carr MH 1997 Synergistic predation, density dependence, and population regulation in marine fish. Science 277, 946–949. (doi:10.1126/science.277.5328.946)

[24] Holbrook SJ, Schmitt RJ 2002 Competition for shelter space causes density-dependent predation mortality in damselfishes. Ecology 83, 2855–2868. (doi:10.1890/0012-9658(2002)083[2855:CFSSCD]2.0.CO;2)

[25] Gaines S, Roughgarden J 1985 Larval settlement rate: a leading determinant of structure in an ecological community of the marine intertidal zone. Proc. Natl Acad. Sci. USA 82, 3707–3711. (doi:10.1073/pnas.82.11.3707) 16593571 10.1073/pnas.82.11.3707 PMC397856

[26] Raimondi PT 1990 Patterns, mechanisms, consequences of variability in settlement and recruitment of an intertidal barnacle. Ecol. Monogr. 60, 283–309. (doi:10.2307/1943059)

[27] Raymundo LJ, Maypa AP 2004 Getting bigger faster: mediation of size-specific mortality via fusion in juvenile coral transplants. Ecol. Appl. 14, 281–295. (doi:10.1890/02-5373)

[28] Linden B, Rinkevich B 2017 Elaborating an eco-engineering approach for stock enhanced sexually derived coral colonies. J. Exp. Mar. Biol. Ecol. 486, 314–321. (doi:10.1016/j.jembe.2016.10.014)

[29] Puill-Stephan E, van Oppen MJH, Pichavant-Rafini K, Willis BL 2012 High potential for formation and persistence of chimeras following aggregated larval settlement in the broadcast spawning coral, *Acropora millepora*. Proc. R. Soc. B 279, 699–708. (doi:10.1098/rspb.2011.1035) 10.1098/rspb.2011.1035 PMC3248722 21752820

[30] Connell JH 1985 The consequences of variation in initial settlement vs postsettlement mortality in rocky intertidal communities. J. Exp. Mar. Biol. Ecol. 93, 11–45. (doi:10.1016/0022-0981(85)90146-7)

[31] Knowlton N 2001 The future of coral reefs. Proc. Natl Acad. Sci. USA 98, 5419–5425. (doi:10.1073/pnas.091092998) 11344288 10.1073/pnas.091092998 PMC33228

[32] Hughes TP, Baird AH, Dinsdale EA, Moltschaniwskyj NA, Pratchett MS, Tanner JE, Willis BL 2000 Supply-side ecology works both ways: the link between benthic adults, fecundity, and larval recruits. Ecology 81, 2241–2249. (doi:10.1890/0012-9658(2000)081[2241:SSEWBW]2.0.CO;2)

[33] Hughes TP, Tanner JE 2000 Recruitment failure, life histories, and long-term decline of Caribbean corals. Ecology 81, 2250–2263. (doi:10.1890/0012-9658(2000)081[2250:RFLHAL]2.0.CO;2)

[34] Arnold SN, Steneck RS, Mumby PJ 2010 Running the gauntlet: inhibitory effects of algal turfs on the processes of coral recruitment. Mar. Ecol. Prog. Ser. 414, 91–105. (doi:10.3354/meps08724)

[35] Brock RE 1979 Experimental study on the effects of grazing by parrotfishes and role of refuges in benthic community structure. Mar. Biol. 51, 381–388. (doi:10.1007/BF00389216)

[36] Vermeij MJA, Smith JE, Smith CM, Thurber RV, Sandin SA 2009 Survival and settlement success of coral planulae: independent and synergistic effects of macroalgae and microbes. Oecologia 159, 325–336. (doi:10.1007/s00442-008-1223-7) 19050932 10.1007/s00442-008-1223-7

[37] Heyward AJ, Smith LD, Rees M, Field SN 2002 Enhancement of coral recruitment by *in situ* mass culture of coral larvae. Mar. Ecol. Prog. Ser. 230, 113–118. (doi:10.3354/meps230113)

[38] Edwards AJ, Guest JR, Heyward AJ, Villanueva RD, Baria MV, Bollozos IS, Golbuu Y 2015 Direct seeding of mass-cultured coral larvae is not an effective option for reef rehabilitation. Mar. Ecol. Prog. Ser. 525, 105–116. (doi:10.3354/meps11171)

[39] Suzuki G, Arakaki S, Suzuki K, Iehisa Y, Hayashibara T 2012 What is the optimal density of larval seeding in *Acropora* corals? Fish. Sci. 78, 801–808. (doi:10.1007/s12562-012-0504-6)

[40] Graham EM, Baird AH, Willis BL, Connolly SR 2013 Effects of delayed settlement on post-settlement growth and survival of scleractinian coral larvae. Oecologia 173, 431–438. (doi:10.1007/s00442-013-2635-6) 23525803 10.1007/s00442-013-2635-6

[41] Oliver JK, Willis BL 1987 Coral-spawn slicks in the Great Barrier Reef—preliminary observations. Mar. Biol. 94, 521–529. (doi:10.1007/BF00431398)

[42] Therneau TM 2012 Coxme: mixed effects Cox models. *R Package version*.

[43] Heyward AJ, Negri AP 1999 Natural inducers for coral larval metamorphosis. Coral Reefs 18, 273–279. (doi:10.1007/s003380050193)

[44] Mundy CN 2000 An appraisal of methods used in coral recruitment studies. Coral Reefs 19, 124–131. (doi:10.1007/s003380000081)

[45] Bates D, Maechler M, Bolker B, Walker S 2015 Fitting linear mixed-effects models using lme4. J. Stat. Softw. 67, 1–48. (doi:10.18637/jss.v067.i01)

[46] Hothorn T, Bretz F, Westfall P 2008 Simultaneous inference in general parametric models. Biom. J. 50, 346–363. (doi:10.1002/bimj.200810425) 18481363 10.1002/bimj.200810425

[47] R Development Core Team. 2016 R: a language and environment for statistical computing. 3.3.2 ed Vienna, Austria: R Foundation for Statistical Computing.

[48] Korner-Nievergelt F, Roth T, Felten S, Guelat J, Almasi B, Korner-Nievergelt P 2015 Bayesian data analysis in ecology using linear models with R, BUGS and Stan. Amsterdam, The Netherlands: Elsevier.

[49] Anderson MJ, Gorley RN, Clarke KR 2008 PERMANOVA+ for PRIMER: guide to software and statistical methods. Plymouth, UK: PRIMER-E.

[50] Jones G, Almany G, Russ G, Sale P, Steneck R, van Oppen M, Willis B 2009 Larval retention and connectivity among populations of corals and reef fishes: history, advances and challenges. Coral Reefs 28, 307–325. (doi:10.1007/s00338-009-0469-9)

[51] Connolly SR, Baird AH 2010 Estimating dispersal potential for marine larvae: dynamic models applied to scleractinian corals. Ecology 91, 3572–3583. (doi:10.1890/10-0143.1) 21302829 10.1890/10-0143.1

[52] Feng M, Colberg F, Slawinski D, Berry O, Babcock R 2016 Ocean circulation drives heterogeneous recruitments and connectivity among coral populations on the North West Shelf of Australia. J. Mar. Syst. 164, 1–12. (doi:10.1016/j.jmarsys.2016.08.001)

[53] Graham EM, Baird AH, Connolly SR, Sewell MA, Willis BL 2013 Rapid declines in metabolism explain extended coral larval longevity. Coral Reefs 32, 539–549. (doi:10.1007/s00338-012-0999-4)

[54] Whalan S, Wahab MAA, Sprungala S, Poole AJ, de Nys R 2015 Larval settlement: the role of surface topography for sessile coral reef invertebrates. PLoS ONE 10, e0117675 (doi:10.1371/journal.pone.0117675) 25671562 10.1371/journal.pone.0117675 PMC4324781

[55] Doropoulos C, Roff G, Visser M-S, Mumby PJ 2017 Sensitivity of coral recruitment to subtle shifts in early community succession. Ecology 98, 304–314. (doi:10.1002/ecy.1663) 27870014 10.1002/ecy.1663

[56] Nozawa Y 2008 Micro-crevice structure enhances coral spat survivorship. J. Exp. Mar. Biol. Ecol. 367, 127–130. (doi:10.1016/j.jembe.2008.09.004)

[57] Nozawa Y 2012 Effective size of refugia for coral spat survival. J. Exp. Mar. Biol. Ecol. 413, 145–149. (doi:10.1016/j.jembe.2011.12.008)

[58] Edmunds PJ, Nozawa Y, Villanueva RD 2014 Refuges modulate coral recruitment in the Caribbean and the Pacific. J. Exp. Mar. Biol. Ecol. 454, 78–84. (doi:10.1016/j.jembe.2014.02.009)

[59] Brandl SJ, Bellwood DR 2016 Microtopographic refuges shape consumer-producer dynamics by mediating consumer functional diversity. Oecologia 182, 203–217. (doi:10.1007/s00442-016-3643-0) 27147547 10.1007/s00442-016-3643-0

[60] Christiansen NA, Ward S, Harii S, Tibbetts IR 2009 Grazing by a small fish affects the early stages of a post-settlement stony coral. Coral Reefs 28, 47–51. (doi:10.1007/s00338-008-0429-9)

[61] Doropoulos C, Ward S, Marshell A, Diaz-Pulido G, Mumby PJ 2012 Interactions among chronic and acute impacts on coral recruits: the importance of size-escape thresholds. Ecology 93, 2131–2138. (doi:10.1890/12-0495.1) 23185875 10.1890/12-0495.1

[62] Gallagher C, Doropoulos C 2017 Spatial refugia mediate juvenile coral survival during coral–predator interactions. Coral Reefs 36, 51–61. (doi:10.1007/s00338-016-1518-9)

[63] Jayewardene D, Donahue MJ, Birkeland C 2009 Effects of frequent fish predation on corals in Hawaii. Coral Reefs 28, 499–506. (doi:10.1007/s00338-009-0475-y)

[64] Gibbs DA, Hay ME 2015 Spatial patterns of coral survivorship: impacts of adult proximity versus other drivers of localized mortality. PeerJ 3, e1440 (doi:10.7717/peerj.1440) 26623193 10.7717/peerj.1440 PMC4662597

[65] Holm ER 1990 Effects of density-dependent mortality on the relationship between recruitment and larval settlement. Mar. Ecol. Prog. Ser. 60, 141–146. (doi:10.3354/meps060141)

