# Supplementary material for: Density-dependent coral recruitment displays divergent responses during distinct early life-history stages
Source: R Soc Open Sci. 2017 May 17;4(5):170082. doi: 10.1098/rsos.170082 (PMC5451816; doi:10.1098/rsos.170082)
Supplement: Figure S1 [file rsos170082supp1.pdf]

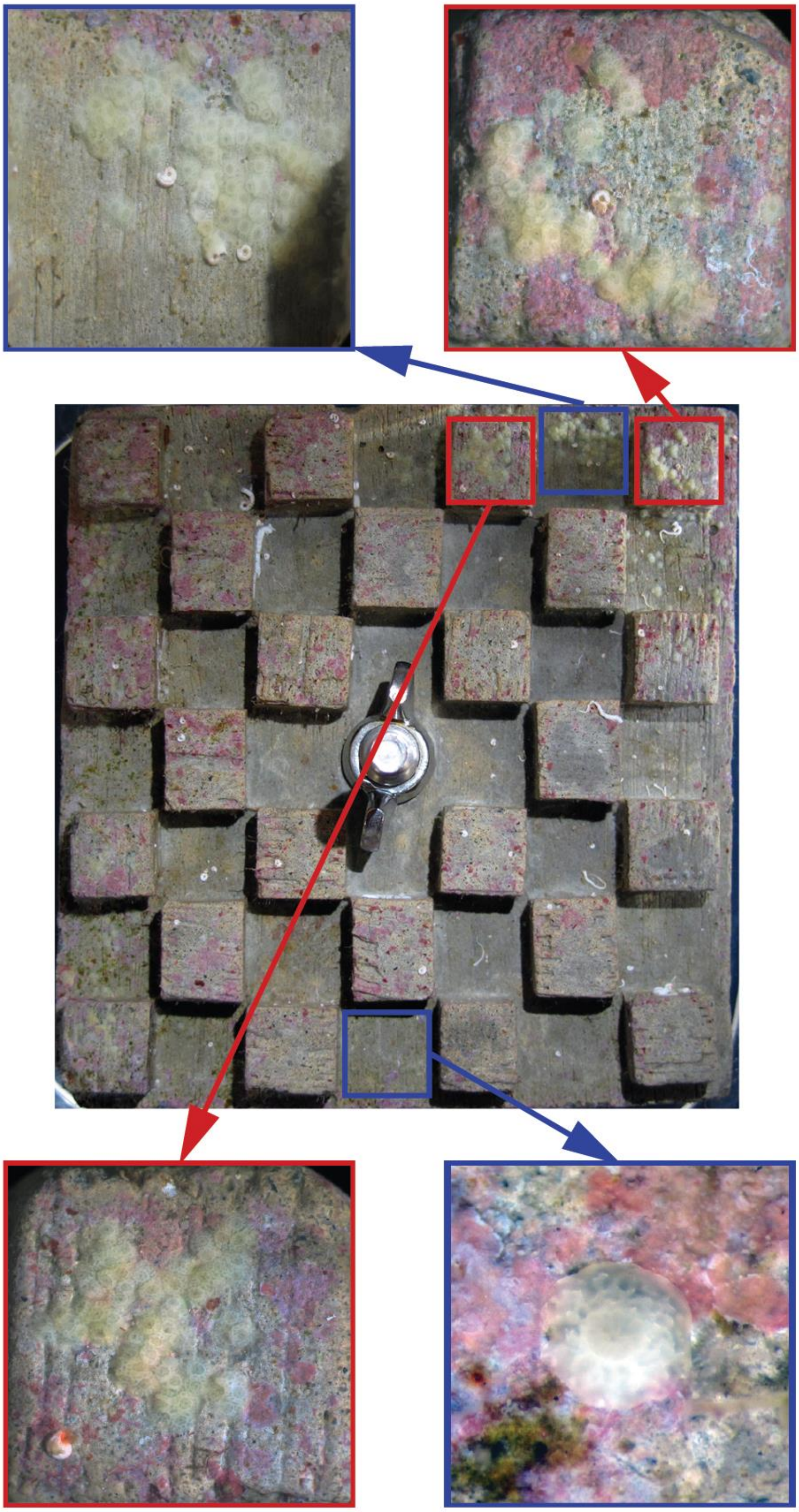

Figure S1 An example custom made settlement tile with newly settled *Acropora millepora* recruits that are singular or aggregated on exposed (red borders) and crevice (blue borders) microhabitats.
