# Supplementary material for: Density-dependent coral recruitment displays divergent responses during distinct early life-history stages
Source: R Soc Open Sci. 2017 May 17;4(5):170082. doi: 10.1098/rsos.170082 (PMC5451816; doi:10.1098/rsos.170082)
Supplement: Figure S2 [file rsos170082supp2.pdf]

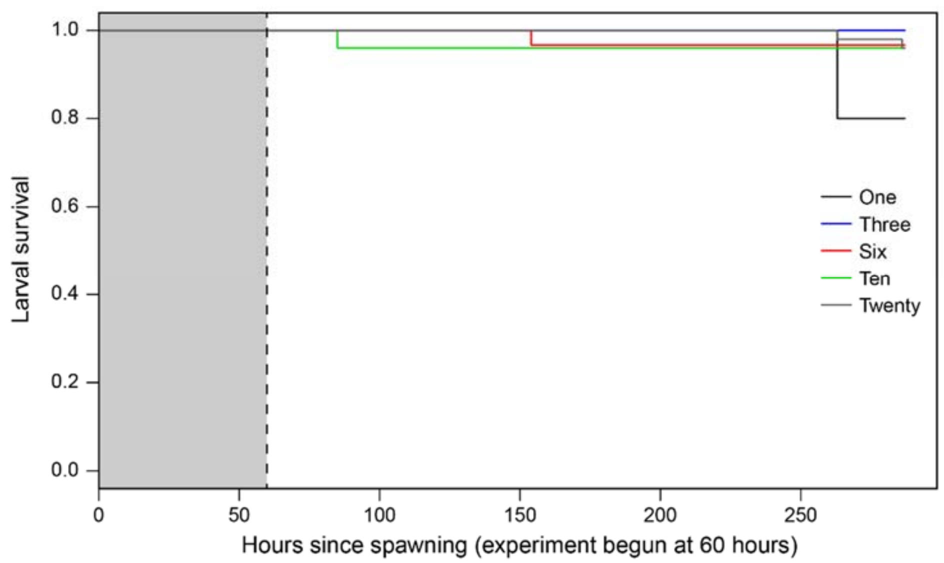

Figure S2 Proportional survival of *Acropora millepora* larvae from 60 to 288 hours following spawning as a function of larval density.
